# Breastfeeding and externalising problems: a quasi-experimental design with a national cohort

**DOI:** 10.1007/s00787-017-1085-9

**Published:** 2017-11-24

**Authors:** Lisa-Christine Girard, Orla Doyle, Richard E. Tremblay

**Affiliations:** 1 School of Public Health, Physiotherapy, and Sports Science, University College Dublin, Dublin, Ireland; 2 Geary Institute for Public Policy, University College Dublin, Dublin, Ireland; 3 School of Health in Social Science, Clinical Psychology, University of Edinburgh, Medical School, Teviot Place, Edinburgh, EH8 9AG UK; 4 School of Economics, University College Dublin, Dublin, Ireland; 5 Research Unit on Children’s Psychosocial Maladjustment (GRIP), Université de Montreal, Montréal, Canada; 6 Departments of Pediatrics and Psychology, Université de Montreal, Montréal, Canada

**Keywords:** Conduct problems, Hyperactivity, Breastfeeding, Longitudinal cohort study, Quasi-experimental approach

## Abstract

Evidence from correlational studies supporting the benefits of breastfeeding on children’s externalising problems is mixed. Quasi-experimental approaches can help in better understanding possible ‘effects’. We aimed to investigate the longitudinal impact of breastfeeding on externalising problems from childhood into adolescence. Participants included ~ 5000 full-term children, from the Growing Up in Ireland Child Cohort. Externalising problems (conduct problems and hyperactivity) were assessed using both the parent and teacher versions of the Strengths and Difficulties Questionnaire when children were age 9 and 13. Maternal reports were used to collect retrospective information on breastfeeding. Propensity score matching, and adjusting for multiple testing were used to compare the average treatment effects for children who were breastfed. Post matching results revealed statistically significant reductions in hyperactivity at age nine, using both maternal and teacher reports (difference score − 0.48, 95% CI − 0.85, − 0.11; and − 0.51, 95% CI − 0.90, − 0.12, respectively), for children who were breastfed between 6 and 12 months, but not thereafter. These effects were not maintained at age 13. Moreover, no effects of breastfeeding on conduct problems were found at any age, regardless of duration, using either maternal or teacher report. While some benefits of breastfeeding were found, compatible with a temporary modest reduction in hyperactivity, related to being breastfed for 26–50 weeks, these results must be viewed in the context of reliance of propensity score matching on observable characteristics. Additionally, our results are suggestive of a potential non-linear dose–response of breastfeeding on hyperactivity.

**Electronic supplementary material:**

The online version of this article (10.1007/s00787-017-1085-9) contains supplementary material, which is available to authorized users.

## Introduction

Breastfeeding has long been associated with better health, immunological advantages, and cognitive outcomes for children from infancy through early adulthood [1–4]. More recently, a growing interest in the potential benefits of breastfeeding on children’s behavioural development has emerged. While breastfeeding and externalising behaviours have been less well-understood, multiple hypotheses regarding the potential mechanisms for associations have been put forth including psychological mechanisms, neurological brain development, and genetic risk. For example, mother–infant bonding via early skin-to-skin touch, eye contact, and cradling during breastfeeding can create more secure attachment in infants [5, 6], independent of shared environment and genetic factors [7], consequently reducing the risk of future externalising problems. Relatedly, higher maternal responsiveness to child needs, marked by greater activation in the superior frontal gyrus, insula, precuneus, striatum, and amygdala, along with increased oxytocin response [8, 9], observed in mothers who breastfeed, have been associated with better child adjustment and reduced risk of externalising problems [10].

Brain development may be another mechanism for the proposed link between breastfeeding and externalising behaviour. The nutrients found in breast milk, in particular the long-chain polyunsaturated fatty acids, have been shown to positively impact on white matter growth, and abnormalities in white matter have been implicated in studies with children who exhibit conduct problems [11] and hyperactivity [12]. Further, white matter growth is particularly critical during the first 2 years, [13] which may support a linear dose–response association of longer durations of breastfeeding. It has also been suggested that breastfeeding may protect against the development of externalising behaviour for children who have an elevated genetic risk [14]. Research on both conduct problems and hyperactivity have consistently revealed a strong genetic link for predisposition to these behaviours [e.g., 15], making increasingly attractive a better understanding of the environmental factors that may protect against their emergence.

Despite the plausibility of the above-mentioned hypotheses, support for the link between breastfeeding and externalising problems across studies has been mixed. Breastfeeding has been associated with reduced externalising behaviours in some studies [16–19] but not in others [20, 21]. Moreover, the issue of causality remains open due to the high reliance on observational studies. For instance, mothers who breastfeed typically have higher levels of education and engage in less risky prenatal behaviours (e.g., smoking) [e.g., 22]. These same characteristics are also associated with children’s behavioural outcomes [23, 24]. Given these differences, lack of randomisation violates statistical assumptions of equivalence between groups, that is between children who were breastfed and children who were not. This raises the possibility that any benefits regarding behavioural outcomes within observational studies may be attributable to selection bias and unobserved confounding that have not been adequately controlled for, rather than to breastfeeding per se.

Indeed, statistical adjustment for confounding due to selection bias (i.e., mothers self-select into breastfeeding and mothers who breastfed may provide more optimal environments) often results in smaller and/or statistically insignificant ‘effects’. Identifying ‘effects’ without the use of randomised control trials is challenging. The best evidence to date on the effects of breastfeeding and children’s externalising behaviour stems from a cluster-randomised breastfeeding promotion trial, conducted in the Republic of Belarus (PROBIT) [21]. As Kramer et al. [21] note, while no statistically significant differences between treatment and control groups on children’s behavioural outcomes were found under the intention to treat analysis, it is possible that the differences between the two groups, regarding the intensity and duration of breastfeeding, were not large enough to detect any effects. This may particularly be the case for behavioural outcomes given that some previous studies have supported a linear dose–response association [25, 26]. Moreover, the PROBIT trial examined behavioural outcomes using dichotomised scores of the Strengths and Difficulties Questionnaire (i.e., abnormal scores in the upper 85th percentile), which have a strong predictive validity of future clinical diagnosis [27]. It is possible that clinical levels of externalising behaviour problems are less amenable to environmental protective factors, such as breastfeeding, particularly regarding shorter durations and partial breastfeeding.

Given the ethical challenges of randomised trials regarding breastfeeding, the use of quasi-experimental statistical approaches, such as propensity score matching, provides the next best approach to understanding any potential ‘effects’ of breastfeeding on children’s behavioural outcomes. Only a few studies to date have used quasi-experimental approaches while examining breastfeeding and behavioural outcomes [e.g., 16, 17], and results from these studies are suggestive of modest effects.

### Objectives

We examined the potential effects of breastfeeding on children’s externalising behaviour from late childhood into adolescence using a quasi-experimental statistical approach, to address the inherent methodological challenges within observational cohort studies. Moreover, we examined whether the benefits of breastfeeding would differ by duration of breastfeeding. It was hypothesised that children who were breastfed would have lower externalising problems compared with children who were not breastfed, with larger effects observed for children who were breastfed for longer durations.

## Methods

Participants included children and their families enrolled in the Growing Up in Ireland (GUI) Child Cohort. Recruitment occurred in the Republic of Ireland between September 2007 and June 2008 using a two-stage random selection process, (i.e., the National School System and random selection of eligible children within each school). The child level eligibility criterion was being born between November 1st 1997 and October 31st 1998. The total sample included 8570 9-year-old children, with a response rate of 82% at the school level and 57% at the individual level. For a detail description of the GUI child cohort, please see [28]. The current study used data collected when children were age 9 and 13. Eligibility criteria in this study included children who were born full-term and who had complete data on matching variables at age nine (*N* = 6013; 70.2%). Girls represented 51.9% (*n* = 3121). Attrition and missing outcome data over time resulted in *N* = 5342 at age 13. Demographic characteristics of this sample can be found in Table 1. Further, a comparison between families included in the current study and those initially recruited can be found in the online supplement. The Health Research Board’s Research Ethics Committee granted ethics approval, and both written consent and assent were collected from parents/guardians and children prior to all data collection.

**Table 1:** Comparison of family, maternal, child and medical characteristics: child cohort at 9 years

	Never breastfed (*N* = 2853)	Ever breastfed (*N* = 3160)	*p*
Family social class:			≤ 0.001
Professional/managerial	1238 (43.4%)	2083 (65.9%)	
Non-manual/skilled manual	1180 (41.4%)	837 (26.5%)	
Semi-skilled/unskilled	327 (11.5%)	163 (5.2%)	
No valid social class	108 (3.8%)	77 (2.4%)	
Medical card status:			≤ 0.001
Free medical care	539 (18.9%)	383 (12.1%)	
Free general practitioner care	71 (2.5%)	65 (2.1%)	
No free medical care	2243 (78.6%)	2712 (85.8%)	
Maternal parenting:			0.024
Authoritative	2230 (78.2%)	2493 (78.9%)	
Authoritarian	89 (3.1%)	135 (4.3%)	
Permissive	474 (16.6%)	482 (15.3%)	
Neglectful	60 (2.1%)	50 (1.6%)	
Paternal parenting:			0.001
Authoritative	1935 (67.8%)	2223 (70.3%)	
Authoritarian	185 (6.5%)	250 (7.9%)	
Permissive	579 (20.3%)	527 (16.7%)	
Neglectful	154 (5.4%)	160 (5.1%)	
Maternal age:			≤ 0.001
< 33 years	424 (14.9%)	251 (7.9%)	
34–38 years	761 (26.7%)	703 (22.2%)	
39–43 years	1048 (36.7%)	1304 (41.3%)	
> 44 years	620 (21.7%)	902 (28.5%)	
Maternal education:			≤ 0.001
Less than high school diploma	714 (25.0%)	250 (7.9%)	
High school diploma	1108 (38.8%)	801 (25.3%)	
College diploma	636 (22.3%)	884 (28.0%)	
University degree	267 (9.4%)	734 (23.2%)	
Professional/graduate degree	128 (4.5%)	491 (15.5%)	
Resident spouse/partner (yes):	2637 (92.4%)	2975 (94.1%)	0.008
Maternal employment status:			≤ 0.001
Employed	1572 (55.6%)	1909 (60.4%)	
Student	13 (0.5%)	51 (1.6%)	
Unemployed/state training	40 (1.4%)	38 (1.2%)	
Home duties/retired	1208 (42.3%)	1138 (36.0%)	
Long-term sickness/disability	20 (0.7%)	24 (0.8%)	
Maternal ethnicity (Irish):	2593 (90.9%)	2483 (78.6%)	≤ 0.001
Smoking during pregnancy (yes):	794 (27.8%)	427 (13.5%)	≤ 0.001
Drinking during pregnancy (yes):	1000 (35.1%)	1396 (44.2%)	≤ 0.001
Delivery mode (Caesarean):	494 (17.3%)	518 (16.4%)	0.479
Child birth weight (< 2500 g-yes):	40 (1.4%)	38 (1.2%)	0.495
Visit to the NICU (yes):	303 (10.6%)	289 (9.1%)	0.055
Child sex (girl):	1502 (52.6%)	1619 (51.2%)	0.274
Siblings living in dwelling (yes):	2660 (93.2%)	2972 (94.1%)	0.195

Medical card cover is a means-tested card issued by health services on the basis of financial need. There are two tiers of medical card cover: ‘free medical care’, which includes visits to general practitioners plus prescriptions and ‘Free general practitioner care’, which excludes prescriptions

### Measures

Children’s behaviours were assessed using both the parent version of the Strengths and Difficulties Questionnaire (SDQ) at ages 9 and 13 and the teacher version at age 9 only. The conduct problems (e.g., ‘often fights with other children or bullies them’) and the hyperactivity (e.g., restless, overactive, cannot stay still for long’) scales were used. Both scales are comprised of five items. Parents and teachers were asked to rate the applicability of the child’s behaviour on a 3-point scale (i.e., *0* = *not true* to *2* = *certainly true*), with possible scores ranging between 0 and 10. The SDQ has been well-validated [29]. Please see Table 2 for the means of maternal- and teacher-rated conduct problems and hyperactivity, along with the correlations between maternal and teacher ratings.

**Table 2:** Bivariate correlations between maternal and teacher SDQ scores and means/standard deviations of children’s outcomes at 9 and 13 years of age

	Conduct problems 9 years (teacher)	Hyperactivity 9 years (teacher)	Mean (SD)	Min–max
Conduct problems 9 years (maternal)	*r* = .22	*r* = .21	1.19 (1.37)	0–10
Hyperactivity 9 years (maternal)	*r* = .23	*r* = .42	2.86 (2.36)	0–10
Conduct problems 9 years (teacher)	–		0.61 (1.28)	0–10
Hyperactivity 9 years (teacher)	*r* = .50	–	2.12 (2.50)	0–10
Conduct problems 13 years (maternal)			1.03 (1.31)	0–10
Hyperactivity 13 years (maternal)			2.41 (2.25)	0–10

All correlations significant at the *p* = < .001 level

Breastfeeding information was collected retrospectively via maternal report when children were age nine. Mothers were asked, “was the child ever breastfed even if only for a short time” and “for how many months or weeks was the study child breastfed”. Support for reliability of recall from 6 to 15 years after breastfeeding occurred has previously been established, with only a slight overestimation bias of 1 week [30, 31]. No information was collected regarding type of breastfeeding in the cohort. Thus, children were grouped into one of four categories: never breastfed (*n* = 2, 853), breastfed up to 25 weeks (*n* = 2, 461), breastfed between 26 and 50 weeks, (*n* = 461), and breastfed 51 weeks or more (*n* = 238), which included partial and exclusive breastfeeding. The three groups of children who were breastfed were treated as mutually exclusive and compared against those who were not breastfed.

Confounders and self-selection into breastfeeding have been suggested to partially account for previous associations found between breastfeeding and child outcomes. Thus, children (breastfed, never breastfed) were matched on 16 factors previously associated with breastfeeding and children’s behavioural outcomes. At the family level, these included social class (professional/managerial, other non-manual/skilled manual, semi-skilled/unskilled, no valid social class), medical card status (free medical care, free general practitioner care, no free medical care), and both maternal and paternal parenting style (authoritative, authoritarian, permissive, neglectful; the Parenting Style Inventory II) [32]. At the maternal level, factors included age (33 years or younger, 34–38 years, 39–43 years, 44 years or older), education (less than high school diploma, high school diploma, college diploma, university degree, graduate degree), partner status (yes/no), employment status (employee, self-employed, farmer, student, state training, unemployed, long-term sickness/disability, home duties/retired), Irish born (yes/no), smoking during pregnancy (yes/no), drinking during pregnancy (yes/no) and type of delivery (vaginal, caesarean). At the child level, factors included birth weight (2500 g or more, yes/no), having neonatal intensive care (yes/no), sex (boy/girl) and sibling status (yes/no).

### Statistical analysis

While experimental designs that randomise breastfeeding conditions are not feasible, statistical techniques such as propensity score matching (PSM) can help overcome selection bias by matching children who were breastfed to those who were not based on their measured characteristics, consequently diminishing differences between groups [33]. Nearest neighbour 1 to 1 matching techniques were used. Nearest neighbour matches groups sequentially, using the closest match between groups once the sample has been randomly ordered. Randomly sorting the sample prior to matching reduces possible bias that could arise from the matching procedure. To ensure the most optimal matches, we imposed a caliper between matches (i.e., a match could only occur if the propensity score fell within a tenth of a standard deviation of each other). Given the low frequency of children who were breastfed between 26 and 50 weeks, and 51 weeks or more, we allowed matching with replacement to ensure a better quality of matches [34]. Balance checks were conducted for each model to ensure bias between the groups was substantially reduced. After matching, remaining bias on individual confounders ranged between 0.0 and 17.9% and the overall mean model bias ranged between 2.4 and 8.2%, indicating successful matching [33]. After matching, all families fell within the area of common support, with the exception of one family whilst examining children who were breastfed 51 weeks or more. Support refers to observations being excluded from the analysis due to not finding a match within the specified caliper. We report the average treatment effect on those who were breastfed. All reported results in the tables have been adjusted for multiple testing using the Holm–Bonferroni method. All statistical analyses were conducted using Stata 13 software.

## Results

Prior to matching, children who were breastfed for up to 25 weeks were rated lower on both maternal- and teacher-rated conduct problems and hyperactivity at age 9, and maternal ratings at age 13, compared to children who were never breastfed. These differences were statistically significant. However, after matching none of the differences remained statistically significant. Results are displayed in Table 3. For children who were breastfed between 26 and 50 weeks, statistically significant differences were found between those who were never breastfed and those breastfed between 26 and 50 weeks for both conduct problems and hyperactivity at age 9 (maternal reports), hyperactivity at age 9 (teacher reports), and hyperactivity at age 13 (maternal reports). All results were in the expected direction (i.e., lower conduct problems and hyperactivity for children who were breastfed between 26 and 50 weeks). These statistically significant differences at age nine, for maternal- and teacher-rated hyperactivity, remained statistically significant after matching and correcting for multiple testing. That is, children who were breastfed between 26 and 50 weeks scored between 0.48 and 0.51 points lower than children who were not breastfed, indicative of a small effect. Maternal-reported conduct problems at age nine did not remain statistically significant after matching. Further, maternal-reported hyperactivity at age 13 did not remain statistically significant after matching. For children who were breastfed 51 weeks or more, statistically significant differences were found prior to matching on maternal-reported hyperactivity (age 9 and 13) and conduct problems (age 9) only. After matching and correction for multiple testing, no statistically significant differences remained.

**Table 3:** Breastfeeding and children’s externalising behaviours at 9 and 13 years of age: pre- and post-matching results

Up to 25 weeks	Pre matching				Post matching			
	*T*	*C*	Diff (sig.)	SE	*T*	*C*	Diff (sig.)	SE
Conduct problems 9 years: maternal	1.12	1.28	**−** 0.16***	0.03	1.12	1.19	− 0.06	0.07
Hyperactivity 9 years: maternal	2.73	3.07	**−** 0.34***	0.06	2.73	2.94	− 0.21	0.11
Conduct problems 9 years: teacher	0.55	0.65	**−** 0.09**	0.03	0.55	0.56	− 0.01	0.06
Hyperactivity 9 years: teacher	1.96	2.30	**−** 0.33***	0.07	1.96	2.12	− 0.15	0.12
Conduct problems 13 years: maternal	0.99	1.07	**−** 0.83*	0.03	0.99	0.96	0.02	0.06
Hyperactivity 13 years: maternal	2.29	2.58	**−** 0.28***	0.06	2.29	2.31	− 0.01	0.11
Between 26 and 50 weeks								
Conduct problems 9 years: maternal	1.06	1.28	− 0.22**	0.07	1.06	1.26	− 0.19	0.11
Hyperactivity 9 years: maternal	2.50	3.07	− 0.57***	0.12	2.50	2.98	− 0.48*	0.19
Conduct problems 9 years: teacher	0.66	0.65	0.01	0.06	0.66	0.60	0.06	0.10
Hyperactivity 9 years: Teacher	1.80	2.30	− 0.50***	0.13	1.80	2.31	− 0.51*	0.20
Conduct problems 13 years	1.02	1.07	− 0.05	0.07	1.02	0.91	0.10	0.11
Hyperactivity 13 years	2.18	2.58	− 0.39**	0.12	2.18	2.26	− 0.07	0.19
51 weeks or more								
Conduct problems 9 years: maternal	0.92	1.28	− 0.36***	0.09	0.92	1.18	− 0.25	0.12
Hyperactivity 9 years: maternal	2.29	3.07	− 0.78***	0.16	2.29	2.66	− 0.36	0.22
Conduct problems 9 years: teacher	0.59	0.65	− 0.06	0.09	0.59	0.52	0.07	0.13
Hyperactivity 9 years: teacher	2.10	2.30	− 0.19	0.17	2.11	1.93	0.17	0.25
Conduct problems 13 years	0.94	1.07	− 0.12	0.09	0.94	0.93	0.00	0.13
Hyperactivity 13 years	2.04	2.58	− 0.53**	0.16	2.05	2.04	0.00	0.22

T denotes ‘treatment’ (breastfed) and C denotes ‘control’ (not breastfed). ‘Diff’ represents the difference in scores between groups. SE refers to the standard errors. For being breastfed up to 25 weeks: N’s at age nine for the treatment group varied between 2458 and 2461 (teacher outcomes, 2369 and 2370) and the control group were between 2850 and 2852 (teacher outcomes, 2766). N’s at age 13 for the treatment group was 2239 and the control group was 2483. For being breastfed between 26 and 50 weeks: N’s at age nine for the treatment group varied between 460 and 461 (teacher outcomes, 445) and the control group varied between 2850 and 2852 (teacher outcomes, 2766). N’s at age 13 for the treatment group was 415 and 2483 for the control group. For being breastfed 51 weeks or more: N’s at age nine for the treatment group varied between 236 and 237 (teacher outcomes, 223) and the control group varied between 2850 and 2852 (teacher outcomes, 2766). N’s at age 13 for the treatment group was 204 and 2483 for the control group

*** Denotes significance at the *p* = < .001 level, ** at the .01 level, * at the .05 level, adjusted for multiple testing

## Comment

Our results contribute to the emerging body of work examining breastfeeding and children’s externalising difficulties in late childhood and adolescence. As the post-matching results are the best estimate of causal effect, we refer only to these. Also note, our results apply only to infants born full-term. In this cohort, we find some statistical support for the positive benefits of breastfeeding in reducing children’s hyperactivity at age nine, as reported by both mothers and teachers, for children who were breastfed between 26 and 50 weeks only. This finding may partially support the duration recommendation of the WHO, suggesting that longer durations, of at least 6 months of breastfeeding, may provide more optimal benefits for children’s development and well-being; in particular relating to their behavioural adjustment (i.e., hyperactivity) into late childhood. We did not find any statistically significant differences for hyperactivity at age nine, in favour of children who were breastfed for extended periods lasting beyond 51 weeks. This may suggest that in the current sample, there was a nonlinear dose–response association of breastfeeding on hyperactivity, similarly to the results of Borra and colleagues, using the Avon Longitudinal Study of Parents and Children [16]. The comparable results between these two studies may be reflective of the comparably low rates of breastfeeding in both Ireland and the UK. Alternatively, this may also reflect the small number of mothers in the current sample, less than 4%, who were still breastfeeding at 51 weeks. With the emergence of more studies using quasi-experimental approaches to examine breastfeeding and behavioural outcomes, replication in cohorts with a greater percentage of women who have breastfed for extended periods (i.e., beyond 12 months) may help in our understanding of whether the association with dose–response for behavioural outcomes is indeed non-linear.

Given that no information was collected on direct breastfeeding versus expressed milk, we were unable to further test the exact mechanism responsible for the reduced hyperactivity (i.e., the physical contact occurring during breastfeeding that helps to create more secure attachment or the nutrients found in breast milk which may impact on reduced hyperactivity via white matter growth). Alternatively, the idea of reverse causation is another potential explanation which merits consideration. In particular, it may be that difficult infants, who display early symptoms of hyperactivity, may be more challenging to breastfeed, especially for longer durations. Studies that are better able to disentangle the question of mechanisms are warranted and would help to advance the current state of knowledge. However, while statistically significant differences between children breastfed between 26 and 50 weeks and those who were never breastfed were found, the effect sizes for both maternal- and teacher-rated hyperactivity at age nine were small. Further, the results did not hold at age 13. This would suggest that these benefits were not maintained into adolescence, a time when environmental factors, such as peer deviance, can exert a more influential role on behavioural problems [35].

While the strengths of this study include the use of a large national developmental dataset from Ireland, a country with one of the lowest breastfeeding rates in Europe [36], the use of a multi-informant approach, coupled with a quasi-experimental approach for minimising selection bias, the use of repeated measures of behaviour, and a larger number of matching confounders compared to other studies using PSM, there remains notable limitations. First, despite previous support for the reliability of breastfeeding recall [25, 26, 37], bias may still be present. Relatedly, given the time elapse between breastfeeding and when mothers were retrospectively asked to recall on their breastfeeding engagement, we categorised breastfeeding into ‘less than 6 months, 26–50 weeks, and 51 weeks or more’ rather than looking at shorter intervals of breastfeeding. In the same vein, given the lower reliability of recall regarding the timing of introduction to complementary solids/liquids, exclusivity was not examined in the current study. There was also no data collected on the method of feeding (i.e., expressed breast milk, direct breastfeeding), impeding our ability to better understand the specific mechanism responsible for the observed benefits. Finally, while the use of PSM is an innovative statistical approach designed to minimise selection bias, PSM is only able to match groups on observable characteristics. It remains possible that unobservable characteristics which impact on both the decision to breastfeed and child outcomes contribute to the statistically significant benefits of breastfeeding found in the current study, which was not accounted for. For example, we had no information on maternal IQ, maternal personality, and postpartum maternal mental health, all of which may be potential contributing characteristics. Future studies would do well to build upon the current work through the inclusion of additional matching variables related to these maternal characteristics. Despite these limitations, our findings add to the current literature by providing some statistical support for the positive impacts of breastfeeding, for at least 6 months, on children’s behavioural development into late childhood, as reported by both mothers and teachers. In a practical context, these advantages remain modest given the small magnitude of effect. Regardless, it should be acknowledged that our results do not take away from any of the numerous medical benefits of breastfeeding afforded to both mothers and infants.

## Electronic supplementary material

Below is the link to the electronic supplementary material.

Supplementary material 1 (DOCX 113 kb)

## Abbreviations

GUI: Growing Up in Ireland
PSM: Propensity score matching
SDQ: Strengths and Difficulties Questionnaire
WHO: World Health Organization
RCT: Randomized control trial
