# Supplementary material for: Breastfeeding and externalising problems: a quasi-experimental design with a national cohort
Source: Eur Child Adolesc Psychiatry. 2017 Nov 24;27(7):877–84. doi: 10.1007/s00787-017-1085-9 (PMC6013516; doi:10.1007/s00787-017-1085-9)
Supplement: Supplementary file 1 — Supplementary material 1 (DOCX 113 kb) [file 787_2017_1085_MOESM1_ESM.docx]

**Supplementary Table 1** *Family, Maternal, Child and Medical Characteristics of Excluded and Include Families: Child Cohort at Nine Years*

|  | Excluded Sample  (N = 2,555) | Current Sample  (N = 6,013) | *p* |
| --- | --- | --- | --- |
| Family social class: |  |  | ≤ 0.001 |
| Professional/managerial | 1,168 (46%) | 3,321 (55%) |  |
| Non-manual/skilled  manual | 865 (34%) | 2,017 (34%) |  |
| Semi-skilled/unskilled | 249 (10%) | 490 (8%) |  |
| No valid social class | 267 (10%) | 185 (3%) |  |
| Medical card status: |  |  | ≤ 0.001 |
| Free medical care | 722 (28%) | 922 (15%) |  |
| Free general  practitioner care | 74 (3%) | 136 (2%) |  |
| No free medical care | 1,758 (69%) | 4,955 (83%) |  |
| Maternal parenting: |  |  | ns |
| Authoritative | 1,518 (77%) | 4,723 (79%) |  |
| Authoritarian | 83 (4%) | 224 (4%) |  |
| Permissive | 324 (17%) | 956 (16%) |  |
| Neglectful | 43 (2%) | 153 (2%) |  |
| Paternal parenting: |  |  | ns |
| Authoritative | 1,026 (68%) | 4,158 (69%) |  |
| Authoritarian | 97 (6%) | 435 (7%) |  |
| Permissive | 310 (21%) | 1,106 (18%) |  |
| Neglectful | 74 (5%) | 314 (5%) |  |
| Maternal age: |  |  | ≤ 0.001 |
| < 33 years | 431 (17%) | 675 (11%) |  |
| 34-38 years | 595 (23%) | 1,464 (24%) |  |
| 39-43 years | 845 (33%) | 2,352 (39%) |  |
| > 44 years | 684 (27%) | 1,522 (25%) |  |
| Maternal education: |  |  | ≤ 0.001 |
| Less than high school  diploma | 546 (21%) | 964 (16%) |  |
| High school diploma | 789 (31%) | 1,909 (32%) |  |
| College diploma | 603 (24%) | 1,520 (25%) |  |
| University degree | 394 (15%) | 1.001 (17%) |  |
| Professional/Graduate  degree | 223 (9%) | 491 (10%) |  |
| Resident spouse/partner (Yes): | 1,965 (77%) | 5,612 (93%) | ≤0.001 |
| Maternal Employment status: |  |  | ns |
| Employed | 1,453 (58%) | 3,481 (58%) |  |
| Student | 42 (1%) | 64 (1%) |  |
| Unemployed/state  training | 54 (1%) | 78 (1%) |  |
| Home duties/retired | 983 (39%) | 2,346 (39%) |  |
| Long-term sickness/  disability | 17 (1%) | 44 (1%) |  |
| Maternal ethnicity (Irish): | 2,100 (82%) | 5,076 (84%) | 0.022 |
| Smoking during pregnancy (Yes): | 611 (28%) | 1,221 (20%) | ≤ 0.001 |
| Drinking during pregnancy (Yes): | 811 (37%) | 2,396 (40%) | 0.008 |
| Delivery mode (Caesarean): | 626 (25%) | 1,012 (17%) | ≤ 0.001 |
| Child birth weight  (< 2500 grams- Yes): | 295 (12%) | 78 (1%) | ≤ 0.001 |
| Visit to the NICU (Yes): | 604 (24%) | 592 (10%) | ≤ 0.001 |
| Child sex (Girl): | 1,283 (50%) | 3,121 (52%) | ns |
| Siblings living in dwelling (Yes): | 2,063 (88%) | 5,632 (94%) | ≤ 0.001 |

Note: Medical card cover is a means-tested card issued by health services on the basis of financial need. There are two tiers of medical card cover: ‘free medical care’, which includes visits to general practitioners plus prescriptions and ‘Free general practitioner care’, which excludes prescriptions. N in the excluded group varies between 2,215 and 2,555 and due to item missingness.
